# *Bacillus velezensis* strain MBY2, a potential agent for the management of crown gall disease

**DOI:** 10.1371/journal.pone.0252823

**Published:** 2021-06-15

**Authors:** Haifa Ben Gharsa, Meriam Bouri, Amira Mougou Hamdane, Christina Schuster, Andreas Leclerque, Ali Rhouma

**Affiliations:** 1 Laboratory of Protection and Improvement of Genetic Resources of Olive, Olive Tree Institute, Tunis, Tunisia; 2 Faculty of Sciences of Tunis, University of Tunis El Manar, Tunis, Tunisia; 3 Institute for Microbiology and Biochemistry, Hochschule Geisenheim University, Geisenheim, Germany; 4 Department of Biology, Technische Universität Darmstadt, Darmstadt, Germany; 5 Department of Genetics and Bioengineering, Faculty of Engineering, Yeditepe University, Istanbul, Turkey; 6 National Institute of Agronomy of Tunis, University of Carthage, Tunis, Tunisia; 7 Consiglio Nazionale delle Ricerche (CNR), Istituto per la Protezione Sostenibile delle Piante (IPSP), Portici, Italy; Academia Sinica, TAIWAN

## Abstract

The reduction of the use chemical pesticides in agriculture is gaining importance as an objective of decision-makers in both politics and economics. Consequently, the development of technically efficient and economically affordable alternatives as, e.g., biological control agents or practices is highly solicited. Crown gall disease of dicotyledonous plants is caused by ubiquitous soil borne pathogenic bacteria of the *Agrobacterium tumefaciens* species complex, that comprises the species *Agrobacterium fabrum* and represents a globally relevant plant protection problem. Within the framework of a screening program for bacterial *Agrobacterium* antagonists a total of 14 strains were isolated from Tunisian soil samples and assayed for antagonistic activity against pathogenic agrobacteria. One particularly promising isolate, termed strain MBY2, was studied more in depth. Using a Multilocus Sequence Analysis (MLSA) approach, the isolate was assigned to the taxonomic species *Bacillus velezensis*. Strain MBY2 was shown to display antagonistic effects against the pathogenic *A*. *fabrum* strain C58 *in vitro* and to significantly decrease pathogen populations under sterile and non-sterile soil conditions as well as in the rhizosphere of maize and, to a lower extent, tomato plants. Moreover, the ability of *B*. *velezensis* MBY2 to reduce C58-induced gall development has been demonstrated *in vivo* on stems of tomato and almond plants. The present study describes *B*. *velezensis* MBY2 as a newly discovered strain holding potential as a biological agent for crown gall disease management.

## Introduction

*Agrobacterium tumefaciens* is a ubiquitous soil borne pathogen responsible for crown gall disease affecting many species of dicotyledonous plants from almost 100 different families, including woody and herbaceous plants [1]. The disease can be identified by the appearance of tumors or galls of varying size and shape on the lower stem and main roots of the plant. The pathogen represents a serious problem for agriculture all over the world. It is considered as a quality pathogen in European countries and a quarantine pathogen in others.

At a genomic level, the bacterial taxon *A*. *tumefaciens* is highly diverse and has been subdivided into ten “genomovars” representing species equivalents within an *A*. *tumefaciens* species complex [2]. For one of these, namely genomovar G8, the new taxonomic species *Agrobacterium fabrum*, represented by its nomenclatural type strain C58, has been introduced on the basis of comparative genomics studies [3].

As *A*. *tumefaciens* is a genetic parasite, the only control option is to prevent the bacterium from infecting susceptible plants. So far, the management of crown gall disease with soil amendments, soil solarization, pre-plant application of different soil sterilizing agents and different antibacterial compounds has been attempted with variable success [4–8].

Among the biological control agents that have been assessed are the *Agrobacterium radiobacter* (heterotypic synonym: *Agrobacterium rhizogenes*) strains K84 and K1026. Although the non-pathogenic strain *A*. *radiobacter* K84 was successful in biological control, its application gave rise to potential problems as decreasing biocontrol efficiency [9]. Loss of biocontrol capacity is mainly related to conjugal transfer of the resistance plasmid pAg84 from the biocontrol strain to pathogenic agrobacteria [10–12]. In order to avoid plasmid transfer and preserve crown gall biocontrol ability, the genetically modified strain K1026 was engineered [13]. However, this strain remains considered as a genetically modified organism subject to legal restrictions in many countries.

Bacteria belonging to the genus *Bacillus* are known to be producers of antimicrobial activities, are easily mass produced, and studies have been performed to identify antagonistic biocontrol strains for managing plant diseases including crown gall [14]. Bacterial strains belonging to several *Bacillus* species as, e.g., *B*. *subtilis* [15] or *B*. *amyloliquefaciens* [16–19] have been positively evaluated in this respect.

The present study aimed to investigate the crown gall biocontrol potential of the halotolerant *Bacillus velezensis* strain (MBY2) that had been isolated from saline soil in Tunisia. In particular, the effect of strain MBY2 on the survival of *A*. *fabrum* under *in vitro* and *in vivo* conditions and on size and proliferation of crown galls *in planta* were assessed. Moreover, the new strain has been molecular taxonomically characterized using a Multilocus Sequence Analysis (MLSA) approach.

## Material and methods

### Isolation and *in vitro* selection of antagonistic bacteria

Bacteria were isolated from rhizosphere soil of the halophytic plant *Zygophyllum album* from Kerkennah Island, Tunisia (site 1: [34°73’35,8” N; 11°29’80,5” E], site 2: [34°73’40,4” N; 11°30’19,6” E], site 3: [34°74’75,2 N; 11°31’12,5” E]). Bacterial isolates were preliminarily characterized by appearance of colonies and Gram staining. Origin and growth conditions of bacterial reference strains used in this work are listed in the Table 1.

**Table 1:** Bacterial strains used in this study.

Strain designation	Source of isolation, geographic origin	Culture media and growth conditions	Reference
*Bacillus velezensis* MBY2	*Z*. *album* Kerkennah, Tunisa	LBA: Peptone 10 g/l, yeast extract 5 g/l, NaCl 5 g/l, pH = 7.2 at 28°C	this study
*Agrobacterium fabrum* C58	America	YPG: Yeast extract 5g/l; peptone 5g/l; glucose 10g/l; pH = 7.2 at 28°C	
*Agrobacterium fabrum* C58Gm^r^			[20]
*Agrobacterium radiobacter* K84	Australia		
*Agrobacterium radiobacter* K1026			[13]

### Assessment of antagonistic activity *in vitro*

Screening for the antagonistic activity of bacterial isolates with respect to *Agrobacterium* was performed by two alternative methods, namely double layer and agar well diffusion assays, as described by [14]. For the double layer method, a suspension of 10^7^ CFU ml^-1^ of the bacterial isolate to be tested was spot-inoculated onto LB medium. After 48h of incubation at 25°C, the potentially antagonistic strain was exposed to chloroform vapor for 30 min and the plates were left open for 15 min in a laminar flow cabinet. One milliliter *of A*. *fabrum* C58 suspension (10^7^ CFU ml^-1^) was mixed with 3 ml of LBA (0.6% agar) at 45°C and quickly inoculated by spreading on the plates containing the isolate to be tested. Plates were incubated at 25°C and checked after 24-48h for the appearance of inhibition haloes surrounding the putative antagonists’ spots.

For the agar well diffusion method, one milliliter of *A*. *fabrum* bacterial suspension (10^7^ CFU ml^-1^) was mixed with 3 ml of YPG (0.6% agar) at 45°C and quickly spread on plates containing LB medium, in which 4 wells of 6 mm diameter were punched. The 24h pre-culture of MBY2 in LB broth were centrifuged at 15,000 rpm for 30 min to remove cell debris and filtered through a 0.2 μm membrane filter, and then 100 μl of each sample were filled into the wells. Plates were incubated at 25°C for 48h and subsequently examined for inhibition haloes around wells to record their size.

For both methods, assays were performed in three biological replicates of one representative experiment.

### Molecular taxonomic identification of bacterial isolate MBY2

Bacterial isolate MBY2 was molecular taxonomically characterized using as markers the 16S rRNA encoding sequence and four genes encoding the large chaperonin subunit (*groEL)*, phosphoribosyl-aminoimidazole-carboxamide formyltransferase (*purH*), the beta subunit of RNA polymerase (*rpoB*), and the alpha subunit of DNA gyrase *(gyrA*) from a MultiLocus Sequence Analysis (MLSA) scheme that had previously been employed to elucidate molecular taxonomic relationships of the *Bacillus subtilis* species complex [21, 22]. The consistency of results obtained with this MLSA scheme has been corroborated by a phylogenomics approach targeting the *Bacillus* species *B*. *velezensis* and *B*. *amyloliquefaciens* [23].

Genomic DNA was extracted from a liquid culture of isolate MBY2 grown aerobically for 24 h at 28°C using a DNeasy Blood & Tissue Extraction kit Qiagen (Hilden, Germany) according to the standard protocol provided by the manufacturer. PCR amplification of marker genes was performed using the Taq PCR Kit (NEB). PCR and sequencing primers (Table 2) were provided by MWG Eurofins. For all markers, a unified PCR program was used that consisted of initial denaturation at 95°C for 5 min, 35 cycles of denaturation at 95°C for 40 s, annealing at 55°C for 1 min and elongation at 68°C for 2 min, and final elongation at 68°C for 4 min. Quality of PCR products was controlled by horizontal agarose gel electrophoresis. PCR products were purified using the Qiaquick PCR Purification kit Qiagen (Hilden, Germany) and Sanger-sequenced by StarSEQ (Mainz, Germany). Raw sequence data were combined into a single consensus sequence for each marker gene using the MEGA program version 6 [24]. Consensus sequences were used as queries in GenBank database searches using the BlastN algorithm [25, 26].

**Table 2:** PCR primers used in this study.

Marker gene	Primer designation	Primer sequence	Reference
16S rRNA	fD1	5´-AGAGTTTGATCCTGGCTCAG	[27]
	rP2	5´- ACGGCTACCTTGTTACGACTT	
	27F	5´- AGAGTTTGATCCTGGCTCAG	[21]
	1492R	5´- GGTTACCTTGTTACGACTT	
*gyrA*	gyrA-42f	5´-CAGTCAGGAAATGCGTACGTCCTT	
	gyrA-1066r	5´-CAAGGTAATGCTCCAGGCATTGCT	
*rpoB*	rpoB-2292f	5´- GACGTGGGATGGCTACAACT	
	rpoB-3354r	5´- ATTGTCGCCTTTAACGATGG	
*purH*	purH-70f	5´- ACAGAGCTTGGCGTTGAAGT	
	purH-1013r	5´- GCTTCTTGGCTGAATGAAGG	
*groEL*	groEL-550f	5´- GAGCTTGAAGTKGTTGAAGG	
	groEL-1497r	5´- TGAGCGTGTWACTTTTGTWG	

In order to create a reference data set for phylogenetic reconstruction for each MLSA marker, published annotated genome sequences from the complex of *Bacillus* species described as the “Operational Group *Bacillus amyloliquefaciens*” [23], i.e. *B*. *amyloliquefaciens* (taxid 1390), *B*. *velezensis* (taxid 492670), and *B*. *siamensis* (taxid 659243), were searched for orthologous genes using the determined MBY2 marker sequences as query. Genome sequences comprising a single identifiable ortholog for each MLSA marker were retained for downstream analysis. The only available MLSA marker sequence *(rpoB*) from the *B*. *siamensis* nomenclatural type strain was added to the marker specific reference set. There were no genome sequence data available from the *B*. *velezensis* type strain. Orthologs from the *B*. *subtilis* subsp. *spizizenii* (taxid 96241) genome served as outgroup sequences.

MBY2 marker genes and identified reference sequences were codon-aligned using the CLUSTAL W function [28] as implemented in the MEGA 6 software package. Moreover, for comprehensive analysis of the information content of the four protein-encoding genes, a codon-aligned concatenation of the MLSA marker sequences was created. The Tree-Puzzle 5.2 software [29] was used to estimate data set specific parameters as nucleotide frequencies, the percentage of invariable sites, the transition/transversion ratio and the alpha-parameter for the gamma-distribution-based correction of rate heterogeneity among sites [30].

For phylogenetic reconstruction from nucleotide sequence alignments, the most appropriate models of DNA sequence evolution were chosen according to the rationale outlined by [31]. Phylogenies were reconstructed i) from p-distance matrices with the Neighbor Joining (NJ) method as implemented in the MEGA 6 software tool under pair-wise deletion of alignment gaps and missing data or ii) using the Maximum Likelihood (ML) method as implemented in the PhyML software tool [32] using the Hasegawa-Kishino-Yano (HKY) model of nucleotide substitution [33] under the assumption of a gamma-distribution-based model of rate heterogeneity [34] allowing for eight rate categories. Tree topology confidence limits were explored in non-parametric bootstrap analyses over 1,000 pseudo-replicates [30].

### Assessment of antagonistic activity in bulk soil and rhizospheres

The *A*. *fabrum* C58Gm^r^ strain obtained from a previous study [35] was co-inoculated with strain MBY2 (1: 1) into sterile and unsterile bulk soil as well as the tomato and maize rhizosphere by simple irrigation of the soil and the seedlings with the bacterial suspension to the field capacity as described by [35]. Briefly, sterile and non-sterile bulk soil (50% peat and 50% sand) were co-inoculated with C58Gmr strain and MBY2 strain (1: 1) at a rate of 10^8^ CFU/g of soil as final concentration of bacterial suspensions. During the incubation at 25°C, soil samples were taken once per week and their appropriate serial dilutions were transferred onto YPG and YPG + 30 μg/ml gentamycin medium to determine respectively the total bacterial population and the proportion of the C58Gm^r^ strains. Assays were performed in three independent experiment, each with three biological repetitions per treatments.

To follow the growth of the C58Gm^r^ strain in the tomato (*Solanum lycopersicum*) and maize (*Zea mays*) rhizospheres in the presence of the antagonistic strain MBY2, seedlings of tomato and maize pre-germinated on agar-agar to 1mm radicle length were transferred to tubes with pre-inoculated soil by the two bacterial strains. Three tubes were used for each treatment and plant. After 30 days of growth, samples were taken from rhizospheric soil and assessed as described above.

### Assessment of antagonistic activity on gall-proliferation

The antagonistic activity of *B*. *velezensis* strain MBY2 *in planta* was carried on both tomato plants (*Solanum lycopersicum* cv Heinz) and almond trees (*Prunus amygdalus* var Mazzetto/GF). Tomato and almond stems were wounded with a scalpel (1–1,5cm) and first inoculated with the pathogenic *A*. *fabrum* C58 suspension (10^6^ CFU/ml). After several minutes, the same site was inoculated with the antagonistic MBY2 suspension (10^6^ CFU/ml). *A*. *radiobacter* biocontrol strain K84 and distilled sterile water were used as positive and negative control treatments, respectively. The effect of bacterial inoculation on the plant was evaluated for up to 70 d post-inoculation visually and by determining gall size and weight [14]. Assays were performed in three independent experiments, each with three biological replicates per treatment.

### Statistical analysis

Statistical analyses were performed using ANOVA test of variance by SPSS software (version 20). Significance of mean differences was determined using the Duncan’s test, with a significance level of 5% (*P* = 0.05). Molecular analysis, including bootstrapping statistics for phylogenetic tree topologies, was carried out using the MEGA 6 software package and the PhyML software for NJ and ML trees, respectively.

## Results

### Bacterial isolation and *in vitro* antagonistic activity screening

**Fig 1:**
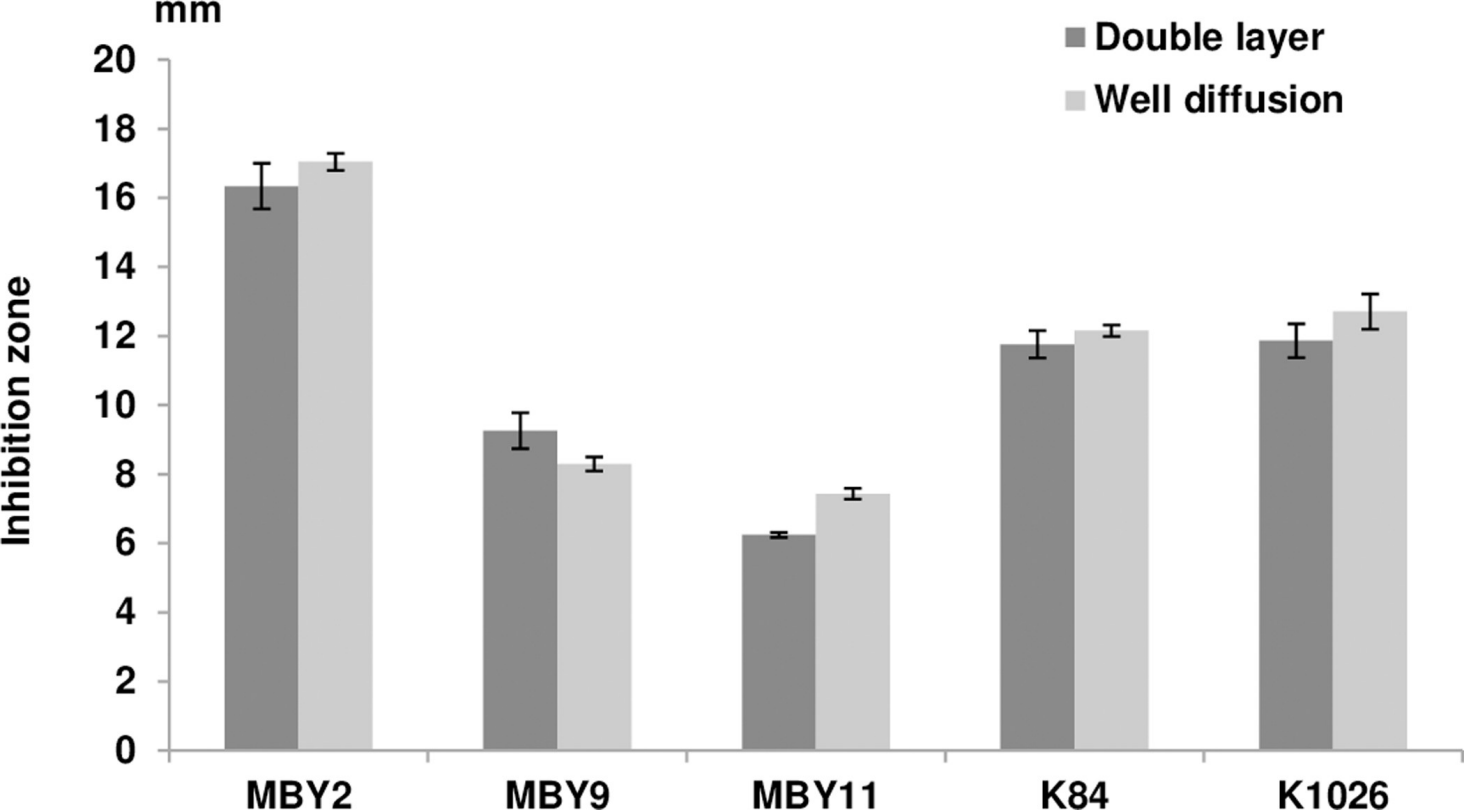
Inhibition of growth of *A*. *fabrum* C58 by bacterial antagonists *in vitro*. Diameters (in mm) of the inhibition zones caused in plates carrying *A*. *fabrum* C58 by the Tunisian *Bacillus* isolates MBY2, MBY9, and MBY11 as well as the commercial biocontrol strains *A*. *radiobacter* K84 and K1026 were determined by both the double layer and the well diffusion method after 48 h at 25°C. Error bars display the standard deviation of three biological replicates performed for each method.

In total, 14 putative *Bacillus* strains were isolated from the rhizosphere of *Z*. *album* from Kerkennah Island, Tunisia, and preliminarily identified as typical Gram positives by staining. *In vitro* screening assays revealed three isolates displaying antagonistic activity towards the *A*. *fabrum* reference strain C58 (Fig 1). Double layer and agar well diffusion assays used to estimate the antagonistic potential gave consistent results for the investigated isolates. Based on the diameters of inhibition haloes, one bacterial isolate termed MBY2 (site 1) was found potentially more efficient than the *A*. *radiobacter* biocontrol strain K84 and its genetically modified derivative K1026 and was, therefore, retained for further investigation.

### Molecular taxonomic identification of isolate MBY2

PCR amplification, sequencing, and raw data assembly revealed a partial sequence (497 bp) of the 16S rRNA encoding gene of isolate MBY2. This sequence was found identical (i.e. displaying 100% nucleotide sequence similarity at 100% sequence coverage) to Genbank database entries representing the *Bacillus* species *B*. *velezensis*, *B*. *amyloliquefaciens*, *B*. *siamensis*, or *B*. *subtilis*. Partial amplification of protein-encoding housekeeping genes gave rise to confirmed consensus sequences for the *rpoB* (813 bp), *groEL* (624 bp), *gyrA* (669 bp), and purH (627 bp) markers. The concatenation of these four marker sequences in the above order generated for more comprehensive data analysis comprised in length 2733 bp. All consensus sequences have been submitted to the Genbank database under accession numbers MT509426 and MT525042—MT525045.

**Fig 2:**
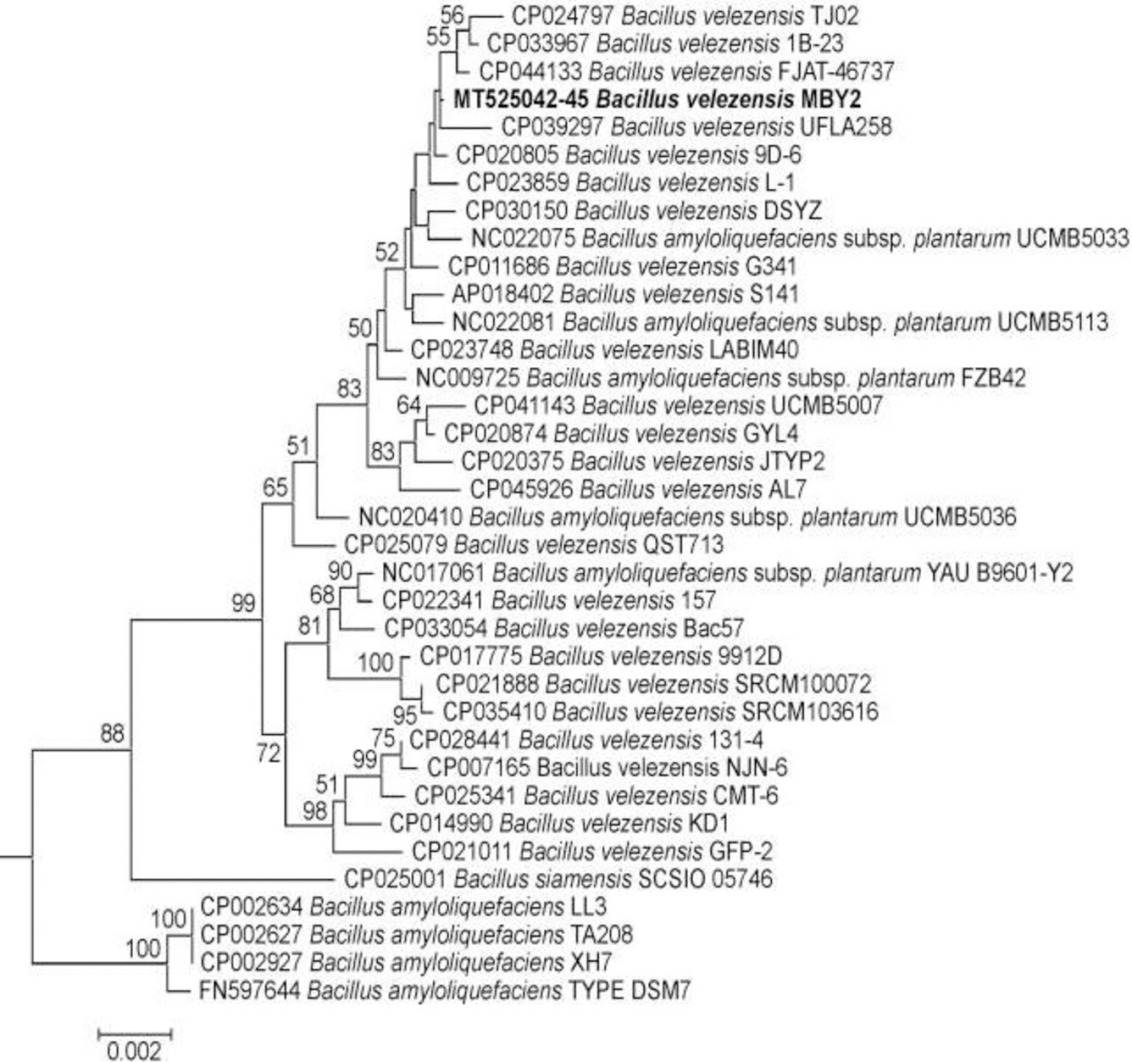
Maximum Likelihood (ML) phylogeny of bacteria belonging to the Operational Group *B*. *amyloliquefaciens* as reconstructed from concatenated *rpoB*, *groEL*, *gyrA*, and *purH* marker sequences. Terminal branches are labelled by GenBank accession number, genus, species and strain designations. The isolate under study is displayed in bold type. Numbers on internal branches indicate bootstrap support percentages. The size bar corresponds to 0.2% sequence divergence along horizontal branches. A concatenation of orthologous sequences from the related bacterium *B*. *subtilis* subsp. *spizenii* served as outgroup to root the tree.

The redundancy-corrected reference data set generated for phylogenetic reconstruction comprised 105 annotated *Bacillus* genomes, i.e. 99 genomes representing the species *B*. *velezensis* or its supposed synonyms *B*. *amyloliquefaciens* subsp. *plantarum* and *B*. *methylotrophicus* in addition to four *B*. *amyloliquefaciens* and one *B*. *siamensis* genomes. In the NJ phylogeny generated for this data set from a concatenation of the four MLSA marker sequences (S1 Fig), the clades representing the species *B*. *velezensis*, *B*. *amyloliquefaciens* and *B*. *siamensis* appeared well separated receiving not less than 99% bootstrap support and the isolate MBY2 was firmly placed within the *B*. *velezensis* clade. Essentially the same result was obtained in both the ML and NJ phylogenies (Figs 2 and S2) generated from concatenated MLSA markers with a reduced reference sequence data set representing the main sub-clades of the full *B*. *velezensis* clade by only 30 genomes. Moreover, in the four NJ phylogenies generated from single MLSA marker sequences across the reduced data set (S3–S6 Figs), isolate MBY2 was located to an internal sub-clade of the *B*. *velezensis* clade, even if bootstrap support for this clade and the concomitant delineation of the species *B*. *velezensis* from *B*. *siamensis*, but not from *B*. *amyloliquefaciens*, appeared low at the single marker level.

### Efficiency of MBY2 in the control of *A*. *fabrum* survival

*B*. *velezensis* strain MBY2 substantially reduced the population density of *A*. *fabrum* C58Gm^r^ in the different inoculation systems used, i.e. in bulk soil as well as in the rhizosphere of tomato and maize plants.

**Fig 3:**
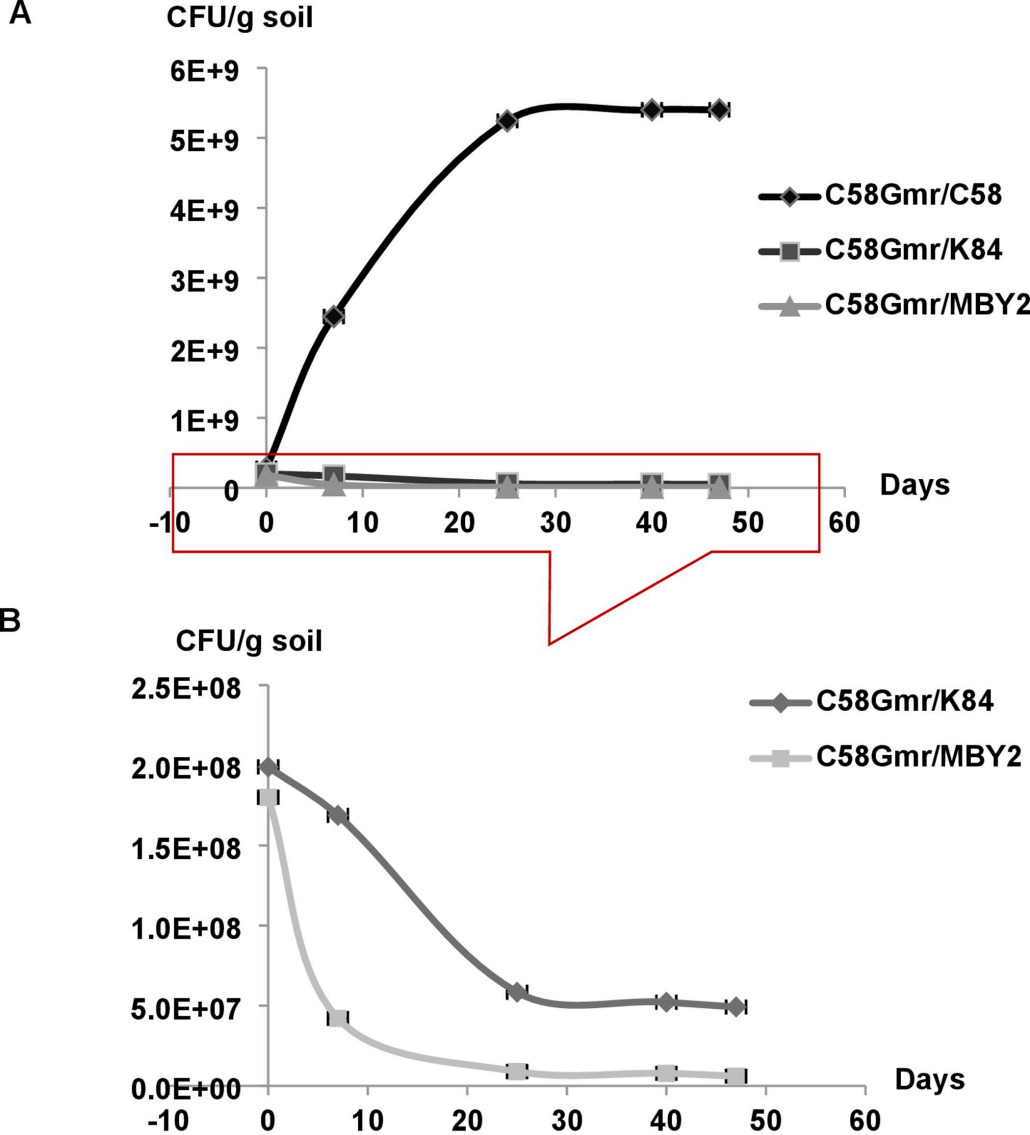
Inhibition of growth of *A*. *fabrum* C58Gm^r^ by *B*. *velezensis* MBY2 in sterile bulk soil. *A*. *fabrum* C58Gm^r^ was co-inoculated (1:1 ratio) with *B*. *velezensis* isolate MBY2, *A*. *radiobacter* K84 as positive and *A*. *fabrum* C58 as negative control at a rate of 10^8^ CFU/g in sterile bulk soil. Numbers of the C58Gm^r^ bacteria were determined by plating onto YPG medium and YPG + 30 μg/ml gentamycin. Data are presented as the average of three biological replicates of one representative experiment. The lower section of part A has been enlarged for better resolution in part B of the figure.

In bulk soil, the number of gentamycin resistant CFU had decreased by app. 75% 7 days and to a very low level 21 days after the addition of strain MBY2. In contrast, the antagonistic effect of the positive control strain K84 appears comparatively both weaker and delayed (Fig 3).

**Fig 4:**
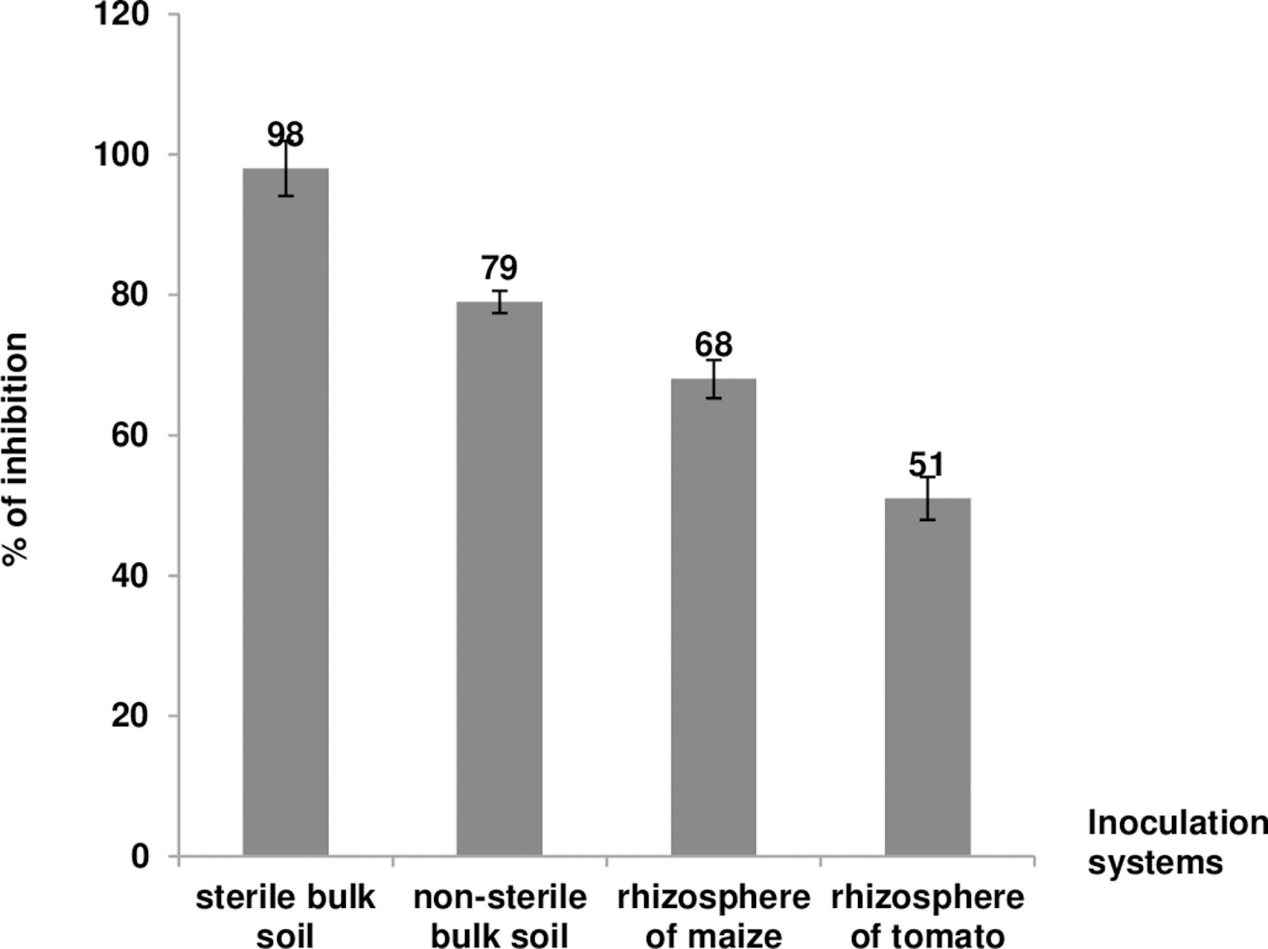
Inhibition of growth of *A*. *fabrum* C58Gm^r^ by *B*. *velezensis* isolate MBY2 in different environments. *A*. *fabrum* C58Gm^r^ was co-inoculated (1:1 ratio) with *B*. *velezensis* MBY2 at a rate of 10^8^ CFU/g in both sterile and unsterile bulk soil as well as the tomato (*Solanum lycopersicum*) and maize (*Zea mays*) rhizosphere. After 30 days, numbers of C58Gm^r^ bacteria were determined by plating onto YPG medium and YPG + 30 μg/ml gentamycin. The proportion (in %) of gentamycin resistant bacteria was calculated with respect to the negative control experiment (C58Gmr/C58). Data are mean ± SD from three biological replicates of one representative experiment.

In the plant rhizosphere, the inhibitory effect of *B*. *velezensis* MBY2 upon multiplication of *A*. *fabrum* C58Gm^r^ 30 d after co-inoculation was more pronounced for maize (68%) than for tomato plants (51%) (Fig 4). However, the antagonistic effect of MBY2 was better in bulk soils than in rhizospheres of both plants and especially under aseptic conditions (sterile bulk soils) (98%) (Fig 4).

### Efficiency of MBY2 in the control of crown gall formation

**Fig 5:**
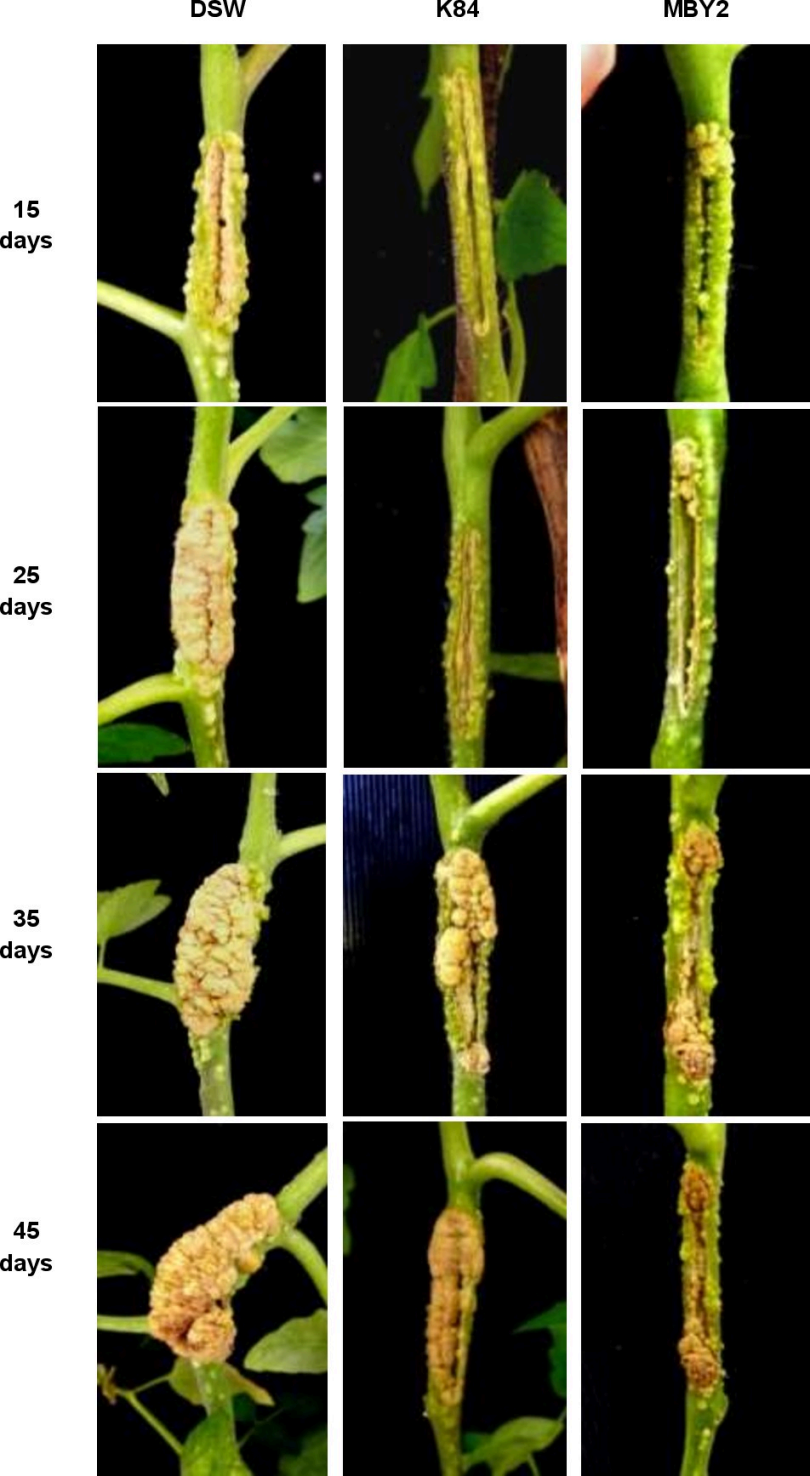
Antagonistic activity *in planta* of *B*. *velezensi*s MBY2 on tomato plants. Stems of tomato (*Solanum lycopersicum* cv Heinz) plants were pre-infected with *A*. *fabrum* C58 and subsequently treated with *B*. *velezensis* isolate MBY2, with *A*. *radiobacter* biocontrol strain K84 as positive or with distilled sterile water (DSW) as negative control. Tumor proliferation was followed for up to 70 days post-inoculation. For each treatment three plants were used in one representative experiment.

**Fig 6:**
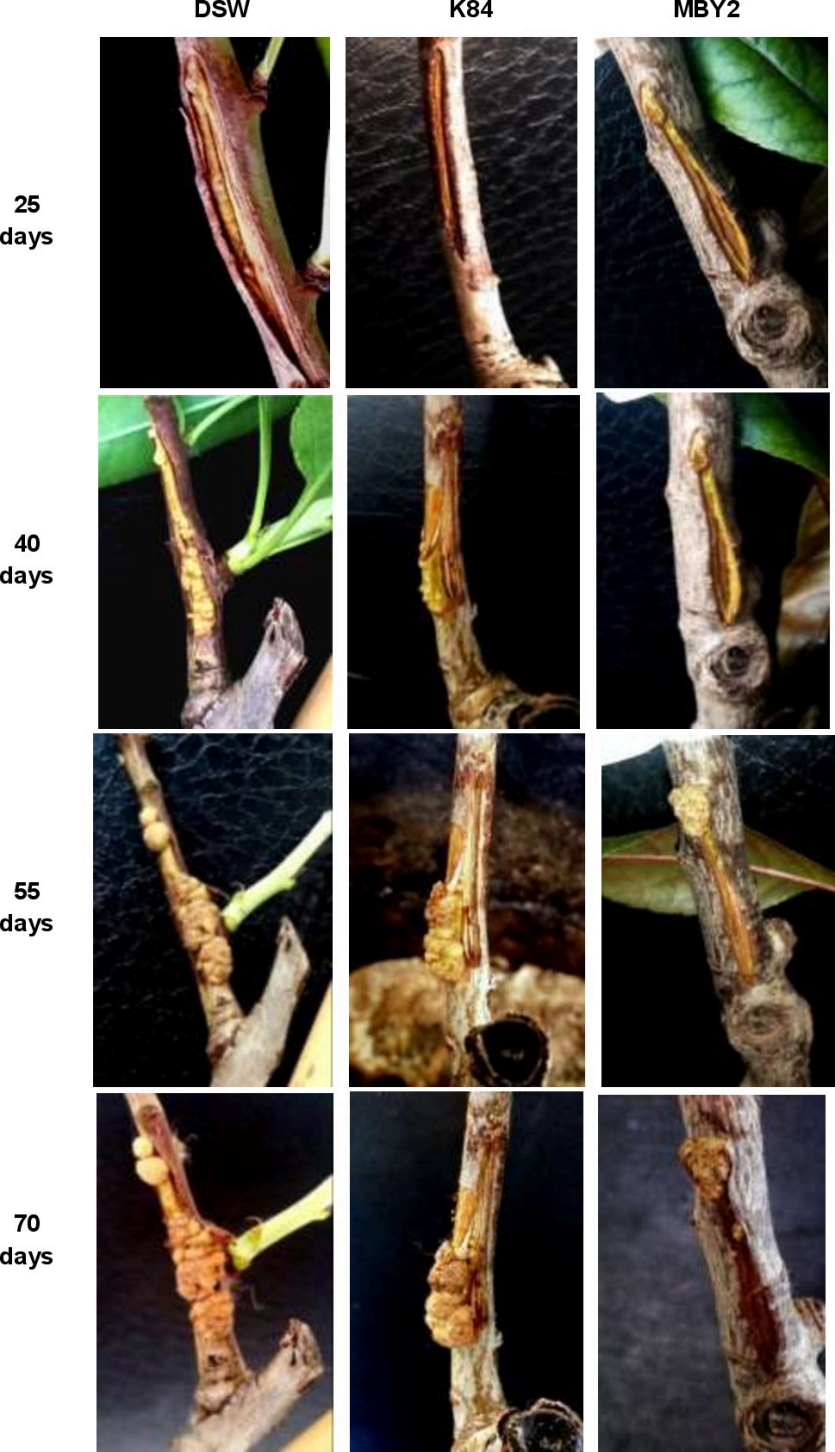
Antagonistic activity *in planta* of *B*. *velezensi*s MBY2 on almond trees. Stems of almond (*Prunus amygdalus* var Mazzetto/GF) trees were pre-infected with *A*. *fabrum* C58 and subsequently treated with *B*. *velezensis* isolate MBY2, with *A*. *radiobacter* biocontrol strain K84 as positive or with distilled sterile water (DSW) as negative control. Tumor proliferation was followed for up to 70 days post-inoculation. For each treatment three plants were used in one representative experiment.

To determine its antagonistic activity, *B*. *velezensis* strain MBY2 was inoculated to tomato and almond stems previously infected with *A*. *fabrum* strain C58 (Figs 5 and 6).

On tomato stems, all inoculated sites began to swell 4 days after inoculation and galls began to appear after 7 d, irrespective of the used treatment, i.e. water, *A*. *radiobacter* K84 and *B*. *velezensis* MBY2. However, 15 d after inoculation, differences in tumor size and proliferation were clearly observed between different treatments. Tumors on plants treated with *A*. *fabrum* C58 and water continuously increased in size until 45 d post-inoculation. In contrast, galls formed on plant stems treated with *A*. *fabrum* C58 and *A*. *radiobacter* K84 gained size more slowly, nodules split progressively at 30 d and had dried out at 40 d post-inoculation. In the case of stems treated with *A*. *fabrum* C58 and *B*. *velezensis* MBY2 the tumor size increased slowly, but proliferation had stopped at 30 d, when tumors darkened and started to dry out. The morphology of the tumors caused on the stems treated with an antagonist strains was similar to that of tumors on negative control plants, but the nodules appeared smaller especially in the plants treated with strain MBY2 (Fig 5).

A similar process of gall formation was observed on stems of almond trees, one of the natural host plants for pathogenic agrobacteria, with a slower tumor progression obviously due to the woody nature of the stems (Fig 6).

**Fig 7:**
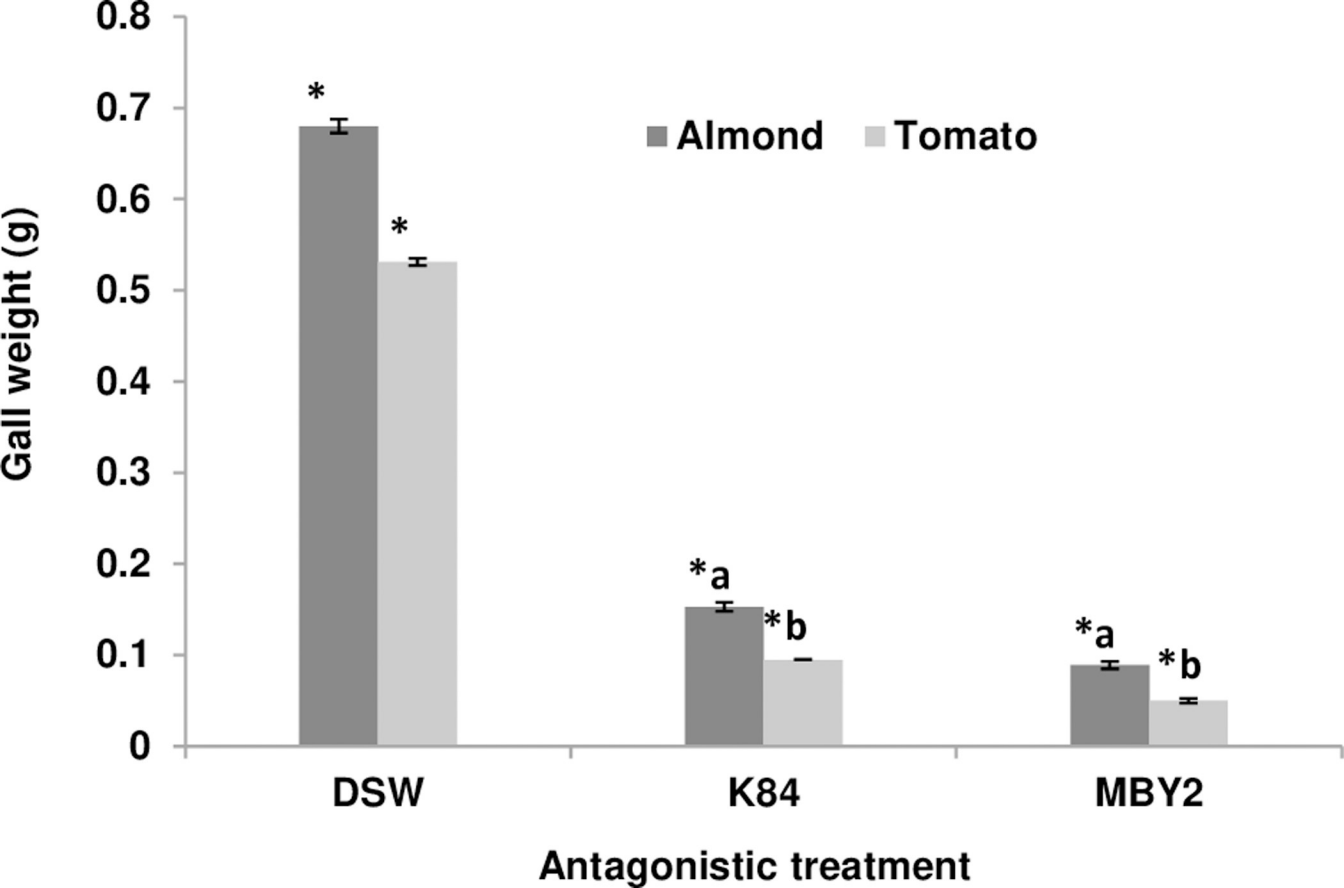
Effect of the antagonistic treatment with *B*. *velezensis* isolate MBY2 on the weight of galls induced by *A*. *fabrum* C58 on tomato and almond plants. Stems of tomato and almond plants were pre-infected with *A*. *fabrum* C58 and subsequently treated with *B*. *velezensis* isolate MBY2, with *A*. *radiobacter* biocontrol strain K84 as positive or with distilled sterile water (DSW) as negative control. Gall weight was determined 70 days after the treatment. Error bars show the variation between three biological replicates. The asterisk (*) designates a significant difference compared to EDS p <0.05. Letters “a” and “b” denote that there was no significant difference between samples with the same letter (p > 0.05).

The effect of treatments on gall weight was very important. In negative controls, the formed tumor mass was 0.68g for almond and 0.531g for tomato, whereas it decreased significantly under treatments with *A*. *radiobacter* K84 (0.153g and 0.095g, respectively) and *B*. *velezensis* MBY2 (0.089g and 0.05g, respectively). Although strain MBY2 led to a strong reduction in gall weight, the effect was not statistically significant as compared to the commercial biocontrol agent K48 used as positive control in this experiment (Fig 7).

## Discussion

Reducing the use of chemical pesticides and inorganic fertilizers are current trends in agriculture in order to enhance environmental quality [36]. Many bacterial genera, including *Bacillus*, are known to be efficient for biocontrol of plant diseases. *Bacillus* bacteria inhabit a large number of different micro-ecosystems and are well known as producers of a wide range of antagonistic compounds of various chemical structures. Soil in general and the plant rhizosphere in particular contain an abundant number of bacilli which play an important role in the control of soil-borne diseases and in plant growth promotion [37].

Bacteria from saline soil have developed strategies of adaptation that allow them to survive under conditions of environmental stress [38]. Rhizobacteria establish a mutually beneficial relationship with their host plant and thereby could be important to improve crop yields. In this context, the antagonistic activity against *A*. *fabrum* C58 of rhizospheric bacteria associated to *Z*. *album*, a halophyte species naturally growing in the sebkha of Kerkennah Island, Tunisia, was assessed.

Among the 14 rhizobacterial isolates from Tunisia, three displayed an antagonistic activity against *A*. *fabrum* C58 *in vitro*. The strain MBY2 showed the best antagonistic activity being more effective to inhibit the growth of strain C58 than the commercial antagonist/biocontrol strains *A*. *radiobacter* K84 and K1026, and was, therefore, retained for further experiments. Determination of the 16S rRNA encoding sequence unequivocally assigned strain MBY2 to the genus *Bacillus* but was found insufficient to differentiate between species within the Operational Group *Bacillus amyloliquefaciens* as had been reported previously by [21] and [23]. Using a concatenation of four protein-encoding gene sequences, isolate MBY2 was identified as belonging to the species *B*. *velezensis* as opposed to the species *B*. *amyloliquefaciens* and *B*. *siamensis*; phylogenetic reconstruction for each of these markers alone was–under insufficient bootstrap support—substantially in line with this assignment, thereby ruling out the possibility that the phylogenetic signal from one of the four MLSA markers had overridden that of the other markers in comprehensive data analysis.

The *B*. *velezensis* type strain CECT-5686 had originally been isolated from brackish water from the river Vélez in Southern Spain [39]. Within the Operational Group *B*. *amyloliquefaciens*, the new species *B*. *velezensis* has been introduced as delineated from *B*. *amyloliquefaciens (sensu stricto)* and *B*. *siamensis* [23, 40] and comprising the synonyms *B*. *amyloliquefaciens* subsp. *plantarum*, *B*. *methylotrophicus* and “*B*. *oryzicola*” [41].

In both *in vitro* and *in vivo* assays *B*. *velezensis* MBY2 was more effective against *A*. *fabrum* than *A*. *radiobacter* K84, the commercial biocontrol agent commonly used for crown gall disease management. The avirulent strain K84 is classified as a true biocontrol agent because it produces antimicrobials as, e.g., agrocin 84, agrocin 434 and ALS84 which enable it to interfere with the growth of virulent agrobacteria in particular [42–44]. However, application of this strain is not always effective as it can induce the resistance of tumorigenic strains from the *A*. *tumefaciens* species complex [44, 45].

*B*. *velezensis* MBY2 clearly reduced the multiplication of *A*. *fabrum* in both bulk soil and the rhizosphere of tomatoes and maize seedlings as compared to *A*. *radiobacter* K84. The antagonistic effect of strain MBY2 was more pronounced in bulk soil and especially in sterile soil. Moreover, the inhibition of *A*. *fabrum* was more pronounced in the maize than in the tomato rhizosphere. This is probably due to the selective role of the plant as in the absence of inhibition by *B*. *velezensis is A*. *fabrum* C58 grows better in the tomato rhizosphere then in that of the non-host plant maize [35].

Results from *in vitro* and *in vivo* assays were in broad agreement with those from *in planta* experiments. The size and proliferation of tumors caused by *A*. *fabrum* C58 on stems of almond and tomato plants was significantly reduced by co-inoculation with *B*. *velezensis* MBY2. In line with our experiments, the efficacy of *Bacillus* sp. strains in reducing gall formation in grapevine and tomato plants had been demonstrated in several previous studies [14–16, 19, 46, 47]. In fact, antagonistic *Bacillus* bacteria can act in at least two not mutually exclusive ways, i.e. either by directly inhibiting the proliferation of microbial phytopathogens or by strengthening plant defense [48]. *B*. *velezensis* MBY2 appears to act directly on pathogenic *A*. *fabrum* bacteria since it was able to inhibit the growth of *A*. *fabrum* strain C58 in *in vitro* and *in vivo* assays in the absence of the plant, presumably by the production of antibacterial compounds.

*Bacillus* bacteria are known to produce a wide spectrum of bioactive compounds as lipopeptides, polyketides and bacteriocins that have been suggested to play an important role in the control of different plant diseases by antagonistic *Bacillus* sp.. Among these compounds, lipopeptides including the families of surfactins, iturines and fengycins have been shown to display potent activities against a wide range of microorganisms [49–52]. Several strains of *B*. *velezensis* as, e.g., strains CR-502T and CR-14b have been characterized by the synthesis of a broad range of lipopeptides with antimicrobial activity [39]. For instance, *B*. *velezensis* strain HYEB5-6 has been reported to be efficient in the control of the anthracnose disease on *Euonymus japonicus* [53], and *B*. *velezensis* strain RC 218 for *Fusarium* control [54]. Generally, the production of antimicrobial substances by *Bacillus* strains starts in the beginning of the exponential growth phase, reaching the maximum in the beginning of the stationary growth phase and has remained at this level throughout the observed period [48], which explains the progressive decrease of the C58 growth curve in the presence of MBY2.

However, *Bacillus* spp. strains have also been identified as plant growth-promoting rhizobacteria such as the BAC03 strain [55] by producing auxin and siderophore [56, 57]. Moreover [58], reported anematicide activity within bacilli strains [58].

## Conclusion

The present study aimed to identify new bacterial antagonist for the biological control of crown gall disease from the microbial communities of saline biotopes in Tunisia. One bacterial isolate from the rhizosphere of *Z*. *album*, that was subsequently identified as *B*. *velezensis* MBY2, was found more efficient than the commercial antagonist strains K84 and K1026 in the inhibition of growth of the plant-pathogen *A*. *fabrum* C58and in the reduction of size and proliferation of tumors induced by the latter. Further analyses such as the investigation of the protective as opposed to the curative potential of strain of strain MBY2, the identification of bioactive molecules and studies of biofilm formation and rhizosphere colonization will be required to better understand the antagonistic mechanism and tap the biocontrol potential of *B*. *velezensis* MBY2.

## Supporting information

S1 Fig Neighbor Joining (NJ) phylogeny of bacteria representing the full range of the Operational Group *Bacillus amyloliquefaciens* as reconstructed from concatenated rpoB, groEL, gyrA, and purH marker sequences. Terminal branches are labelled by GenBank accession number, genus, species and strain designations. The isolate under study is displayed in bold type. Numbers on internal branches indicate bootstrap support percentages. The size bar corresponds to 1% sequence divergence along horizontal branches. A concatenation of orthologous sequences from the related bacterium *Bacillus subtilis* subsp. *spizenii* served as outgroup to root the tree.
(TIFF)

S2 Fig Neighbor Joining (NJ) phylogeny of bacteria belonging to the Operational Group *Bacillus amyloliquefaciens* as reconstructed from concatenated *rpoB*, *groEL*, *gyrA*, and *purH* marker sequences. Terminal branches are labelled by GenBank accession number, genus, species and strain designations. The isolate under study is displayed in bold type. Numbers on internal branches indicate bootstrap support percentages. The size bar corresponds to 1% sequence divergence along horizontal branches. A concatenation of orthologous sequences from the related bacterium *Bacillus subtilis* subsp. *spizenii* served as outgroup to root the tree.
(TIFF)

S3 Fig Neighbor Joining (NJ) phylogeny of bacteria belonging to the Operational Group *Bacillus amyloliquefaciens* as reconstructed from *rpoB* marker sequences. Terminal branches are labelled by GenBank accession number, genus, species and strain designations. The isolate under study is displayed in bold type. Numbers on internal branches indicate bootstrap support percentages. The size bar corresponds to 1% sequence divergence along horizontal branches. The orthologous sequence from the related bacterium *Bacillus subtilis* subsp. *spizenii* served as outgroup to root the tree.
(TIFF)

S4 Fig Neighbor Joining (NJ) phylogeny of bacteria belonging to the Operational Group *Bacillus amyloliquefaciens* as reconstructed from concatenated *groEL* marker sequences. Terminal branches are labelled by GenBank accession number, genus, species and strain designations. The isolate under study is displayed in bold type. Numbers on internal branches indicate bootstrap support percentages. The size bar corresponds to 1% sequence divergence along horizontal branches. The orthologous sequence from the related bacterium *Bacillus subtilis* subsp. *spizenii* served as outgroup to root the tree.
(TIFF)

S5 Fig Neighbor Joining (NJ) phylogeny of bacteria belonging to the Operational Group *Bacillus amyloliquefaciens* as reconstructed from concatenated *gyrA* marker sequences. Terminal branches are labelled by GenBank accession number, genus, species and strain designations. The isolate under study is displayed in bold type. Numbers on internal branches indicate bootstrap support percentages. The size bar corresponds to 1% sequence divergence along horizontal branches. The orthologous sequence from the related bacterium *Bacillus subtilis* subsp. *spizenii* served as outgroup to root the tree.
(TIFF)

S6 Fig Neighbor Joining (NJ) phylogeny of bacteria belonging to the Operational Group *Bacillus amyloliquefaciens* as reconstructed from concatenated *purH* marker sequences. Terminal branches are labelled by GenBank accession number, genus, species and strain designations. The isolate under study is displayed in bold type. Numbers on internal branches indicate bootstrap support percentages. The size bar corresponds to 1% sequence divergence along horizontal branches. The orthologous sequence from the related bacterium *Bacillus subtilis* subsp. *spizenii* served as outgroup to root the tree.
(TIFF)
